# Clast shape-fabric analysis: A comprehensive and efficient methodology to measure particle-orientation data in solid and loose volcaniclastic deposits

**DOI:** 10.1016/j.mex.2023.102519

**Published:** 2023-12-13

**Authors:** L.A. Rodríguez-Sedano, D. Sarocchi, F. Castillo Rivera, G. Moreno-Chávez, M.F. Cerca-Ruiz, J.A. Montenegro-Ríos

**Affiliations:** a CONAHCYT-Instituto de Geología, Universidad Autónoma de San Luis Potosí, Av. Dr. M. Nava No 5 Zona Universitaria, San Luis Potosí 78240, México; b Instituto de Geología/Facultad de Ingeniería-UASLP, San Luis Potosí 78240, México; c Maestría en Ciencias del Procesamiento de la Información, Universidad Autónoma de Zacatecas, Av. Ramón López Velarde 801, Zacatecas 98000, México

**Keywords:** Clast shape-fabric, Particle orientation, Quantitative textural analysis, Vulcanology, Shape-Fabric method

## Abstract

Fabric analysis is essential for understanding the evolution of volcaniclastic deposits. Here we present a comprehensive and efficient methodology, called “Clast shape-fabric analysis,” which is part of the Quantitative Textural Analysis (QTA). This methodology combines high-resolution image analysis techniques with geospatial data processing tools.

The fabric of a deposit refers to the three-dimensional orientation of the particles with respect to space, where the degree of iso-orientation of the major axes of the particles is taken into account. The process begins with the collection of oriented samples in the field. Then, in the laboratory, the samples are processed to obtain high-resolution images. The final stage involves the analysis of these images using the FabricS program, which combines image processing techniques and circular statistics.

An application of the method was made at the Joya Honda Maar in Mexico, where shape-fabric analysis was used to identify the emission centers of pyroclastic materials.

In summary, the “Clast shape-fabric analysis” is a reliable, low-cost and high-potential methodology that can be applied in several geoscientific disciplines and other areas of scientific research.

• New Methodology for shape-fabric analysis is presented.
• The methodology involves field work, laboratory work and image analysis.
• Identification of particle orientations in volcaniclastic deposits.

## Graphical abstract

**Figure:**
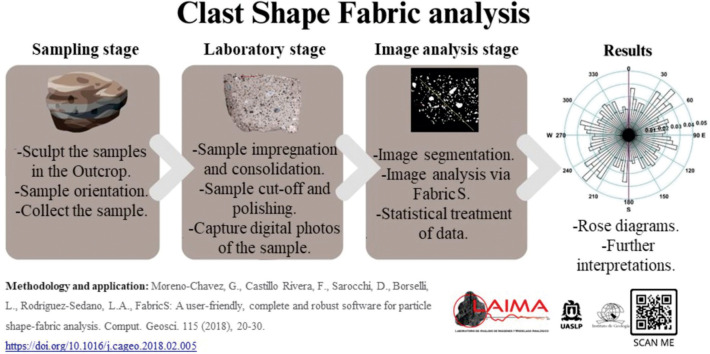

Specifications table

**Table:** 

Subject area:	Earth and Planetary Sciences
More specific subject area:	Volcanology
Name of your method:	Shape-Fabric method
Name and reference of original method:	G. Moreno-Chavez, F. Castillo Rivera, D. Sarocchi, L. Borselli, L. A. Rodríguez-Sedano. FabricS: A user-friendly, complete and robust software for particle shape-fabric analysis. Comput. Geosci. 115 (2018), 20-30. 10.1016/j.cageo.2018.02.005
Resource availability:	FabricS: http://www.laima-uaslp.org/descargas.html

## Method details

### Background

The fabric of a rock or sediment is related to the spatial and geometric configuration of the particles that compose it. In sedimentary rocks, fabric is a property that depends on the depositional environment and can provide information about the directions of the currents at the time of deposition. For this information to be obtainable, some of the particles composing the flowing granular material (crystals, clasts, but also bubbles and voids) must be elongated. In this case we use the term shape-fabric. Shape-fabric refers to the tridimensional orientation and the degree of clast iso-orientation that is recorded in a deposit [1], [2], [3]. The first applications of this textural property to paleo-flow direction go back to the study of turbidity deposits with the purpose of understanding the origin area of these sedimentary materials (e.g., [4], [5], [6]). Later, the same property has been used successfully in volcanology to identify flow directions, vent location, and other characteristics of pyroclastic density currents [1,3,7,[8], [9], [10], [11]] and lahars [12]. Applications of fabric analysis using intercept methods were described by Launeau and Robin [13]. There are various properties that can be studied using shape-fabric, such as determining the polarity of the flow movement, recognizing fluctuations in the flow direction during deposition, and the relationship between the degree of clast iso-orientation and the rheological characteristics of the flow [3]. Shape-fabric can be measured in different ways [14]; those that measure the physical bulk properties of a sample as a whole (bulk methods), and those that measure the orientation of the particles directly, in other words, particle by particle.

**Fig. 1:**
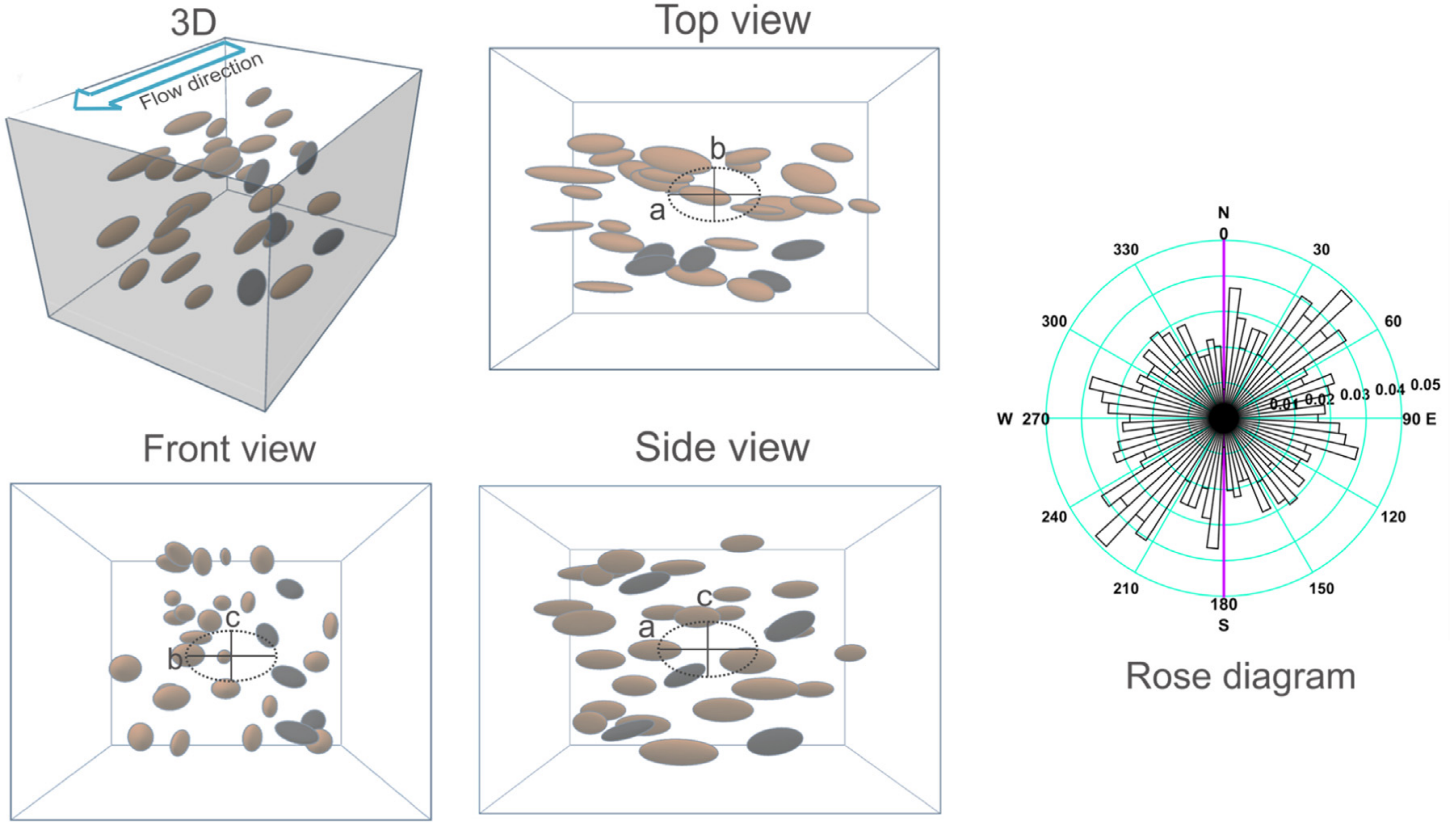
Schematic representation of the quantitative textural analysis (QTA) method focused on the shape-fabric. Three-dimensional view of the particles in an oriented sample.

To the first category belong those methods that measure physical properties such as anisotropy of magnetic susceptivity (AMS) [15], [16], [17], [18], [19], [20], [21], [22], [23], permeability [24], and dielectric intensity [25]. The second category includes the quantitative textural analysis (QTA) method, which is the method described in this paper. QTA applied to a particle's constituent rock or sediment provide geometric data that can be directly related to the paleocurrents, assuming a model for the iso-orientation mechanism (Fig. 1).

In practical applications focused on granular flow directions in a sedimentary environment, clast shape-fabric can be used in different ways, and a variety of different information can be obtained. Data is obtained by analyzing the particles (within the oriented sample) in a horizontal plane or parallel to the bedding planes. For flow polarity it is necessary to measure clast imbrication (by analyzing particles in a vertical plane, oriented parallel to the previously determined flow direction). In some cases, an apparent fabric can also be determined by directly measuring the particles on a vertical wall of an outcrop [26]. The information obtained in this case depends on the outcrop's orientation with respect to the flow, and it can help measure flow polarity (analyzing the imbrication in the outcrop) or to show the presence of erosive channels, lenses, swirls during deposition.

Clast shape-fabric is easy to use, accurate, and low-cost. It consists of taking oriented samples in the field and analyzing them by circular statistics. Here we describe the protocol and methods applied in Cerca-Ruiz [27] and Cerca-Ruiz et al. (in progress), where the method has been used to measure the clast shape-fabric direction and iso-orientation degree, information that allow to construct flow trajectory maps of the pyroclastic deposits of the Joya Honda Maar in San Luis Potosí, Mexico. The same methodology can also be applied to other igneous, sedimentary, and metamorphic rocks. It should be noted that previous versions of this methodology have been applied in the works of Hernandez-Rivas et al. [12] and Zrelak et al. [3], yielding excellent results.

The following sections focus on the methods of sampling and sample processing in the laboratory and describe the protocols of image analysis and data processing.

## Method details

**Fig. 2:**
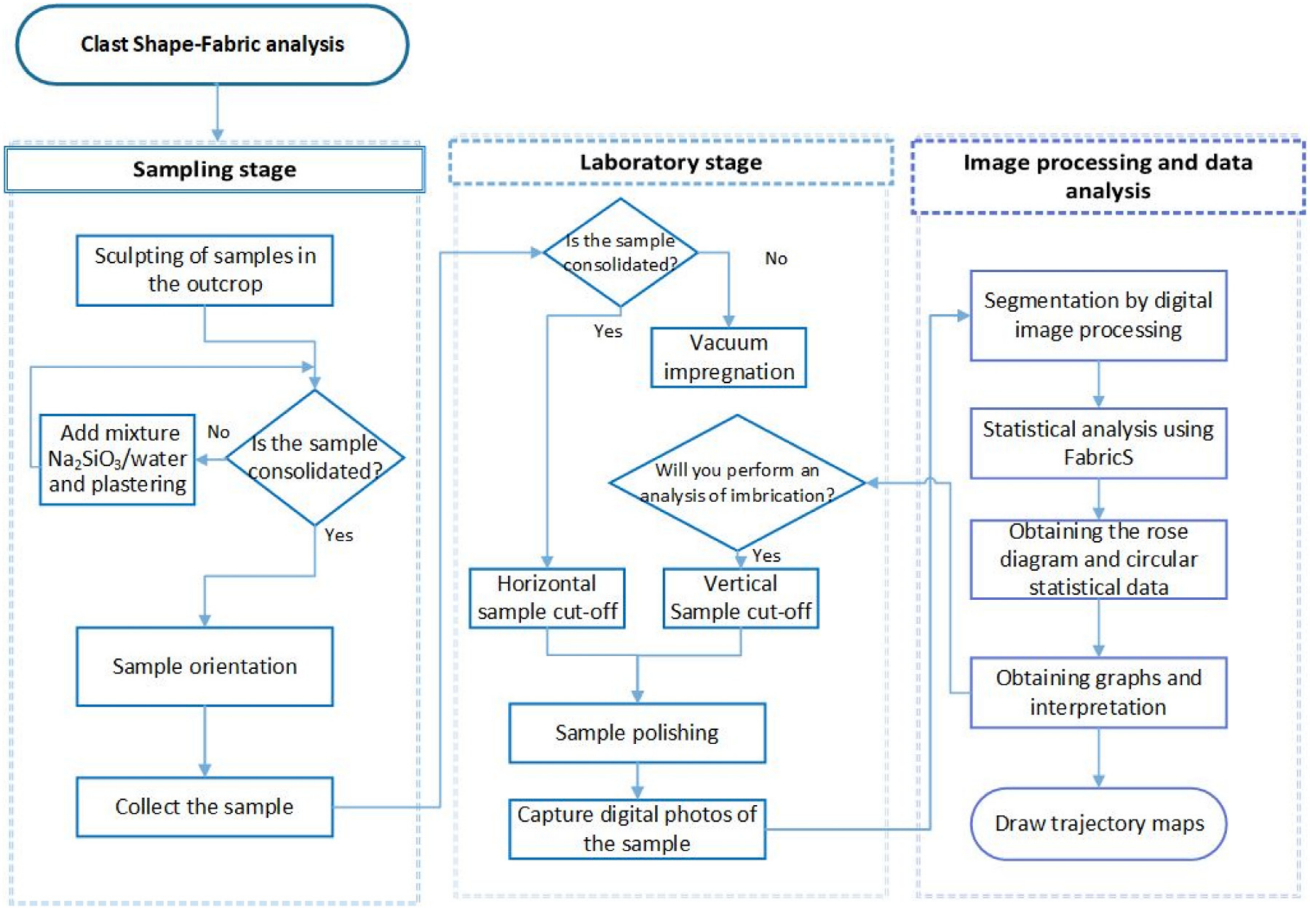
Flowchart indicating the complete protocol to effectively perform the shape fabric analysis.

In order to accurately measure the shape-fabric of a sample from a volcanoclastic deposit, and obtain reliable and reproducible data, it is necessary to follow a specific procedure (Fig. 2).

### Sampling

**Fig. 3:**
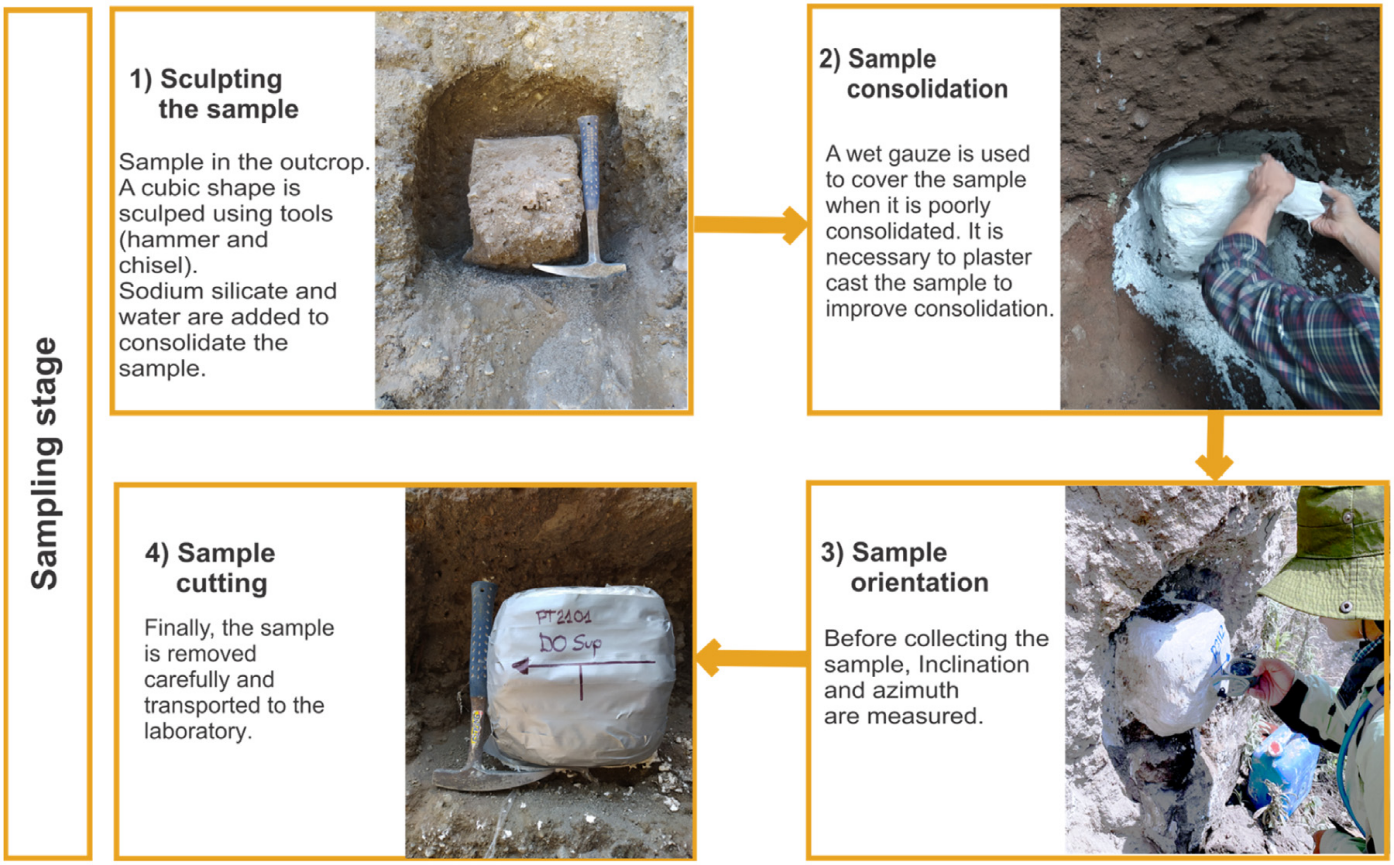
The four main phases of oriented sampling

Location and orientation of samples are among the most important aspects of the process because the accuracy of the data obtained in clast shape-fabric analysis depends on these data. The following workflow summarizes the procedure for obtaining oriented samples in the field (Fig. 3):

1. The unit to be sampled is identified in the outcrop. It is important to note that if more than one stratigraphic unit forms the outcrop, sampling should be carried out in each of them individually depending on the precision required. It is recommended to look for parts of the outcrop surface that are as vertical as possible.
2. With the help of a shovel, hammer and chisel, drill, or any tool that can be used for digging and shaping, a cuboid of approximately 15 × 15 × 15 cm is sculpted in the outcrop. This dimension depends on clast size; the larger the clasts, the larger the sample. If the deposit is unconsolidated, the cuboid should be constantly moistened with a solution of water (75 %) and sodium silicate (25 %) to prevent it from disintegrating while it is being carved out. If the deposit is well consolidated, simply sculpt the cuboid.
3. If the sample is poorly consolidated, it is necessary to stabilize it by means of gauze moistened in a mixture of plaster and water. Moisten the gauze in this mixture and place it on the cuboid until it is covered. Then add more plaster on the cuboid and use a spatula to try to smooth each of its faces as much as possible. Let it dry (approximately 8 hrs).
4. Once the plaster cast has hardened, proceed to orient the sample. With the help of a level, draw a horizontal line on the front face of the cuboid. Draw another line perpendicular to the horizontal line near the bottom of the sample. This line will indicate the direction of the basal part of the sample. Measure the azimuth of the sample by placing a compass on the horizontal line (making sure that the compass is perfectly horizontal, with the help of the level contained in the compass). Next, measure the inclination of the front face of the cube by placing the compass parallel to the vertical line or measuring the inclination with an inclinometer. Finally (if possible), mark an arrow pointing north on the upper or lower face of the cuboid.
5. If the sample is consolidated, draw the corresponding lines on the faces of the cuboid with a marker.
6. Carefully remove the cuboid by detaching the part that is still in contact with the outcrop. This can be done using a hammer and a long chisel or a drill with a long bit. This procedure must be slow and precise to avoid breaking the cuboid.
7. In the case of unconsolidated samples, adding a mixture of sodium silicate (40 %) and water (60 %) to the oriented sample can help to pre-consolidate it (if necessary) to avoid any deformation of the particles inside the cuboid.

### Processing the samples in laboratory

**Fig. 4:**
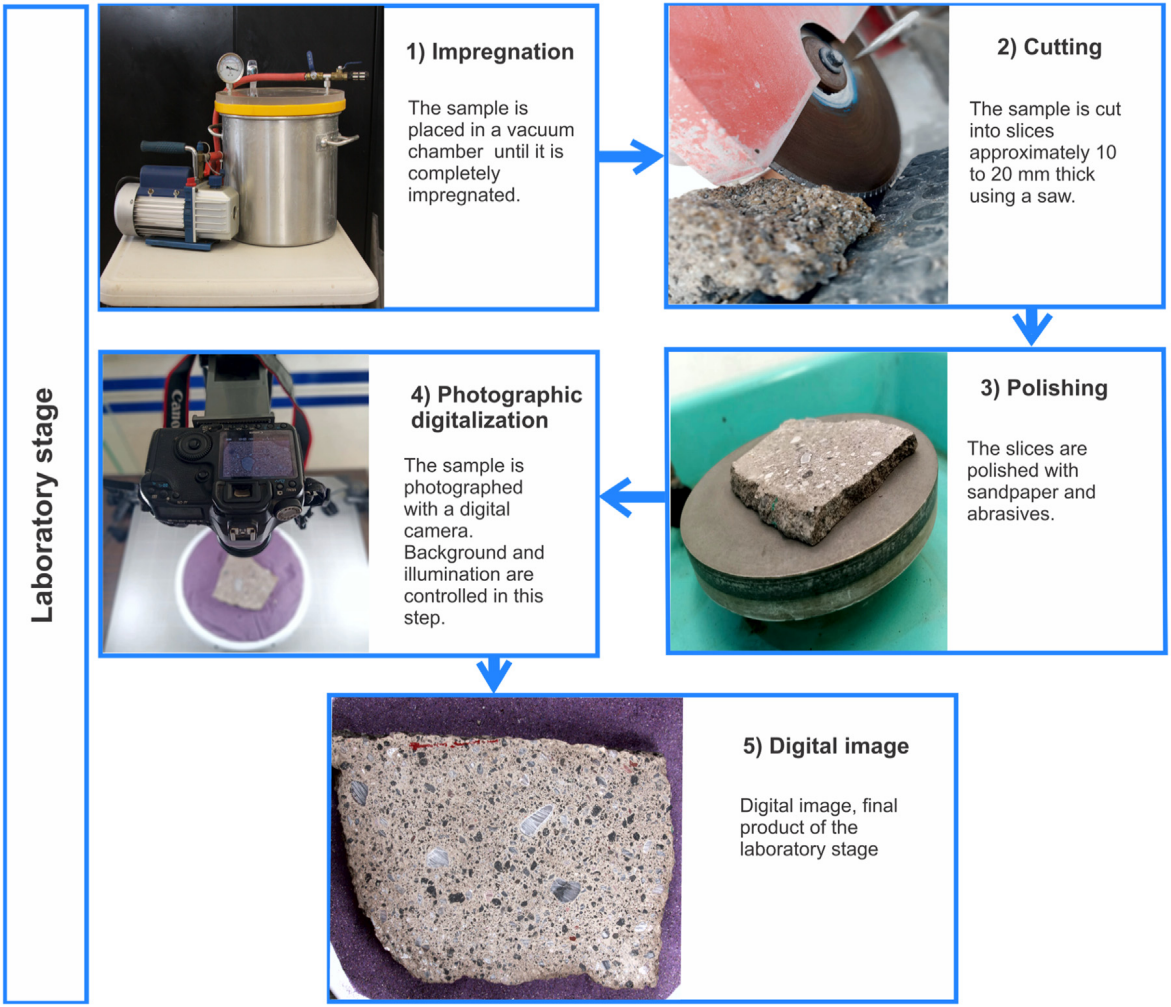
The five main phases of laboratory process.

Once the samples have been oriented and collected in the field, they are carefully transported to the laboratory. The objective of the laboratory procedure is to obtain high-resolution images of horizontal slices of the samples for subsequent image analysis (Fig. 4). The processing of the samples in the laboratory is shown in the following workflow:

1. If necessary, the samples are placed in a vacuum chamber for as long as needed until they are completely impregnated. The time will depend on the type of sample, its degree of consolidation and porosity. It is important to note that with poorly consolidated samples it is recommended not to exceed −0.8 PSI to avoid disintegration.
2. Once consolidated, the sample can be sliced with a saw. A first cut can divide the sample in half in a plane parallel to the vertical. This is so that one half can be processed to analyze the clast shape-fabric and the other half to analyze imbrication of the clasts. Next, a series of cuts are made parallel to the horizontal plane to obtain slices approximately 10 to 20 mm thick.
3. The slices are then polished using sandpaper and abrasives (between 80 and 600 sandpaper grit size) to obtain a smooth finish in which the particles are visible with the greatest possible clarity. On each slice, it is important to mark an arrow indicating the direction of north.
4. Once the slices are polished, they are photographed, placing the north arrow parallel to the vertical side of the photograph. The slice should be placed on a horizontal table and the photograph should be perpendicular to the slice.
5. Once the shape-fabric analysis for flow orientation is finished, the second half of the sample is cut vertically parallel to the flow orientation in order to measure the imbrication. The slab thickness is the same as in Step 2.

### Image analysis and data processing

To measure particle orientation on the photographs taken in the previous phase, a series of procedures needs to be followed. These processes include segmentation of the photograph and subsequent image analysis using the FabricS program [2]. The purpose of the segmentation phase is to obtain an image in which the particles are filled with white while the matrix (particles that are not visible in the photograph) is black. For the analysis of segmented images, the use of the FabricS program is highly recommended. FabricS [2] is a new easy-to-use free software by which it is simple to calculate the shape-fabric of volcano-sedimentary particles. FabricS is the first software dedicated to shape-fabric analysis that combines image analysis processing and circular statistics algorithms. The data obtained by FabricS are the orientation, iso-orientation degree, P-value and rose diagrams of the studied outcrop, verified by rigorous statistical tests such as the Rayleigh test. Both processes (segmentation and image analysis) are detailed in the following workflow:

### Segmentation

The segmentation process consists of separating the matrix and the particles using image editing software. The particles are colored in white and the matrix is colored black in order to obtain binary images. It is recommended that this process be carried out manually in order to minimize the errors and increase the accuracy of the data.

The process of segmentation in Adobe Photoshop is detailed in the following work list; however, there are other free software similar to Photoshop (Ink Scape, Image J, Paint.net) that can be used instead:

1. Input the photograph into Photoshop.
2. Using the “Quick Selection” option, select the sample.
3. In the top menu select the option selection → invert.
4. Using the “quick selection” tool, mark the outline of each of the particles in the photograph. For a better visualization of the finest particles, it is recommended to zoom in. Select all visible particles.
5. From the menu bar choose Image→ Adjustments → Hue/Saturation. In the Hue/Saturation window activate the “Colorize” option and set the following values: Hue= 0, Saturation= 25 and Lightness= 100.

This will assign the color white to the particles.

6. To color the matrix black, in the menu bar select the option selection → Invert. This selects only the matrix. Repeat Step 5, but changing the “Lightness” value to −100. This differentiates the matrix from the particles.

Any editing software that have at your disposal can be used, as long as it can produce an image where the matrix and particles are differentiated from each other.

### Image analysis by FabricS

The main purpose of the overall methodology described in this paper is to obtain the preferential orientation of the particles that make up the analyzed samples. In order to achieve this, we suggest the use of the FabricS program, developed at the LAIMA laboratory of the Universidad Autonoma de San Luis Potosi. FabricS was developed in a MATLAB environment. It is not open source software and currently runs on Windows 7 or higher operating systems. FabricS combines image analysis procedures and circular statistics algorithms. It has an easy-to use graphical interface and basic functions for image processing. The software can process a single image or a set of images. It also allows some parameters to be adjusted, such as the minimum particle size to be considered in the analysis and the elongation of the particles. For more information, we suggest reading the work of Moreno-Chavez et al., [2]. The FabricS user interface contains four sections: file, process, fabric, and export, briefly described below:

### File

The file section allows the user to load binary or color images. This button will open a dialog box where the user can load the binary image first and then the original color image. The process is similar when multiple images need to be loaded.

### Process

The process section has two main tools for processing the image. The first is the complement section, which allows the user to set the background of the image to black and the particles to white if needed. The second tool is the ROI button which enables the user to select a region of interest to analyze instead of the whole image. In addition, the program allows two important parameters to be adjusted, the size threshold and the particle eccentricity threshold. The size threshold can be used to select the minimum size (in pixels) of the particles to be considered in the analysis. The particle eccentricity threshold determines the degree of ellipticity of the particles ranging from 0 to 1, where zero is an indicator of circular particles and 1 indicates very elongated particles. It is important to note that the more circular the shape of the particle, the less orientation information it will yield. Therefore, the eccentricity threshold eliminates all particles that do not provide accurate information due to their round shape. For recommended parameters, it is suggested to see Moreno-Chavez et al., [2]. Once these thresholds have been optimally configured, click on the DO button to perform the analysis.

### Fabric

In the fabric section, the user can define the orientation (north) of the image(s) using the reference option. After that, the user can select the desired statistical parameters (mean, median, or mode). The user must then click on the “stats” button so that the program displays the selected statistics on the image in vector form. The fabric section graphically visualizes the data obtained from the analysis. If the user wishes to export the data, the user must go to the export tab.

### Export

**Fig. 5:**
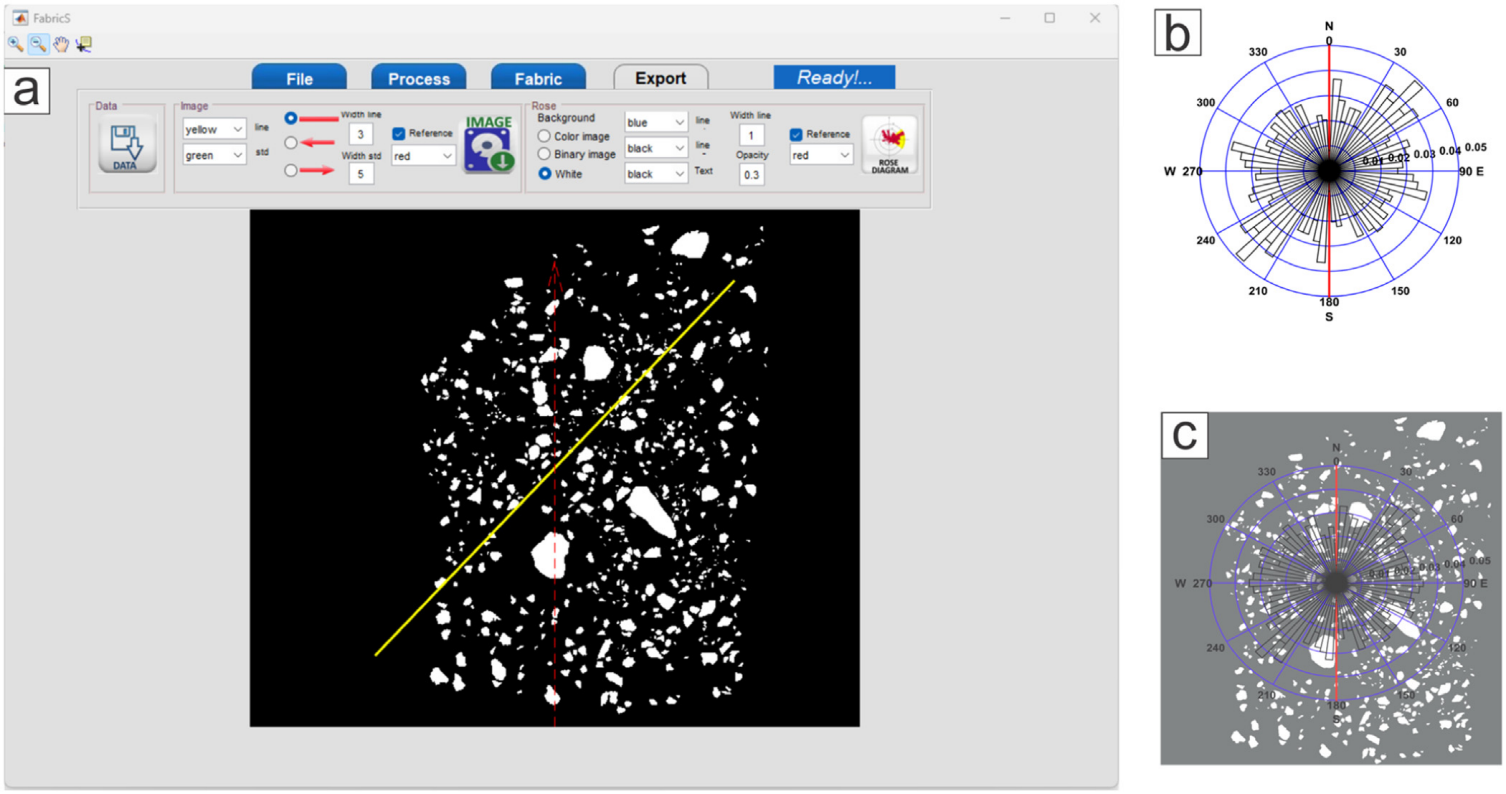
FabricS interface showing each of the tabs and their functions.

In this tab, the user can export the data obtained from the analysis, such as the statistics and the statistical tests that indicate the certainty of the results. The processed images can also be exported. The program may take a few minutes, depending on the processed image(s). Another important product that the program can export is the rose diagram of the data, which graphically shows the iso-orientation of the analyzed particles (Fig. 5).

## Method validation

**Fig. 6:**
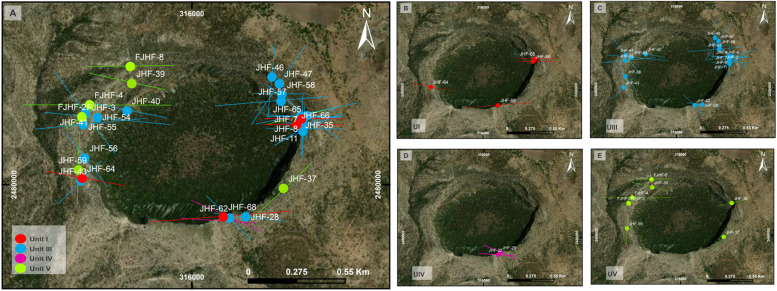
Map of trajectories of the eruptive phases of the Joya Honda. (A) General distribution of the phases. (B) Trajectory map of UI. (C) Trajectory map of unit III. (D) Trajectory map of unit IV. (E) Trajectory map of the UV.

The versatility and potential of the shape-fabric analysis can be demonstrated on a study carried out in Joya Honda Maar (work in progress) located 40 km north of the city of San Luis Potosí in central Mexico. According to previous work, the eruption that created the Joya Honda maar occurred approximately 311 ± 19 ka ago along a fissure and formed a crater 1.3 × 0.9 km and 270 m deep [28]. Five eruptive phases were documented on Saucedo et al. [28], and the emission center was estimated based only by means of stratigraphy. In order to locate the possible center or centers of emission, 77 oriented samples were taken around the crater in the work of Cerca-Ruiz [27] and in Cerca-Ruiz (work in progress) (Fig. 6). All five units were sampled. However, for practical purposes and to expose the effectiveness of the method, only the results obtained from the Unit III are presented below. The complete research work will be presented later in a scientific article.

A total of 38 oriented samples were collected from Unit III following the methodology described in this work. At the same time, a geophysical study (magnetometry) were performed in the maar crater and the surroundings and a ballistic impact study were carried out in the same Unit III.

As a result of the shape-fabric study, a trajectory map was constructed (Fig. 6a) evidencing a possible emission zone in the northern sector of the crater. However, according to the results of particle orientations in all five units another emission center was located at the southern sector of the crater.

An interesting aspect of this study was that the other methodologies (magnetometry and ballistic analysis) yielded similar results. Through magnetometry an anomaly related to a possible conduct was detected at North of the crater and ballistic analysis provide evidence that an emission center probabli was located in the North of the crater. This is in agreement with the results reported by Loera et al. [29] and Saucedo et al. [28].

This case study confirmed that shape-fabric analysis is a reliable methodology that can be used on other similar volcanoes to identify emission centers or source areas of pyroclastic materials or sediments. The potential of this method is vast, as it can be applied in other geosciences such as structural geology ([30], [31], [32]), geophysics [33,34], or sedimentology [35,36], but also in other fields of science such as planetary science [37,38], biostatistics [17,39], and more, due to its simplicity and low cost.

## CRediT authorship contribution statement

**L.A. Rodríguez-Sedano:** Conceptualization, Supervision, Investigation, Validation, Data curation, Writing – original draft, Writing – review & editing. **D. Sarocchi:** Conceptualization, Supervision, Investigation, Writing – original draft, Writing – review & editing. **F. Castillo Rivera:** Software, Validation, Data curation. **G. Moreno-Chávez:** Conceptualization, Software, Validation, Writing – review & editing. **M.F. Cerca-Ruiz:** Validation, Data curation. **J.A. Montenegro-Ríos:** Software, Visualization, Writing – review & editing.

## Declaration of Competing Interest

The authors declare that they have no known competing financial interests or personal relationships that could have appeared to influence the work reported in this paper.

## Data Availability

No data was used for the research described in the article.
